# Head and neck tumor organoid grown under simplified media conditions model tumor biology and chemoradiation responses

**DOI:** 10.1038/s41598-025-88082-5

**Published:** 2025-07-07

**Authors:** Weilin Li, Michiya Nishino, Eric Reed, Dipikaa Akshinthala, Hamdan Ahmed Pasha, Erik S. Anderson, Ling Huang, Hannah Hebestreit, Stefano Monti, Ernest D. Gomez, Scharukh M. Jalisi, Senthil K. Muthuswamy

**Affiliations:** 1 Laboratory of Cancer Biology and Genetics, Center for Cancer Research, National Cancer Institute, National Institutes of Health, Bethesda, MD 20892 USA; 2 Department of Medicine, Beth Israel Deaconess Medical Center, Harvard Medical School, Boston, MA 02215 USA; 3 Department of Pathology, Beth Israel Deaconess Medical Center, Harvard Medical School, Boston, MA 02215 USA; 4 Department of Medicine, Albert Einstein College of Medicine, Bronx, New York 10461 USA; 5 Department of Surgery, Section of Otolaryngology/Head and Neck Surgery, Aga Khan University, Karachi, 74800 Pakistan; 6 Division of Otolaryngology/Head and Neck Surgery, Beth Israel Deaconess Medical Center, Boston, MA 02215 USA; 7 Department of Radiation Oncology, Beth Israel Deaconess Medical Center, Harvard Medical School, Boston, MA 02215 USA; 8 Pancreatic Cancer Center, Henry Ford Health, Detroit, MI 48202 USA; 9 Department of Pharmacology and Toxicology, Michigan State University, East Lansing, MI 48824 USA; 10 Department of Medicine, Computational Biomedicine Section, Boston University Chobanian and Avedisian School of Medicine, Boston, MA 02118 USA; 11 Bioinformatics Program, Faculty of Computing and Data Science , Boston University, Boston, MA 02215 USA; 12 Department of Biostatistics, Boston University School of Public Health, Boston, MA 02118 USA; 13 Department of Otolaryngology/Head and Neck Surgery, Harvard Medical School, Boston, MA 02115 USA

**Keywords:** Biological techniques, Cancer, Cell biology, Genetics

## Abstract

Head and neck squamous cell carcinoma (HNSCC) is a prevalent and often fatal malignancy associated with significant treatment-related toxicity. There is an urgent need for a preclinical model to assess therapeutic options and guide clinical decision-making. To define conditions for establishing patient-derived organoid (PDO) models that faithfully recapitulate morphological, histopathological, and genomic characteristics of HNSCC patients and can predict radiation and chemotherapy responses in patients, PDOs were generated from a group of HNSCC patients. The morphological, histological, mutational, and biological characteristics and treatment responses were evaluated. We demonstrate that the PDOs closely resemble resected tumors from which they were derived with respect to histopathology, differentiation state markers, p16 status, and mutation profiling. We observe patient-to-patient variation in cell proliferation rates. Additionally, they exhibit differential responses to radiotherapy and chemotherapy, which were examined using a cell viability assay. This methodology offers potential for drug screening in a pre-clinical context with the potential to mirror clinical outcomes. Our WNT-free growth conditions maintained the differentiation status of PDOs and enabled rapid assessment of drug response and the development of new models to identify new treatment options for head and neck cancer patients.

**Supplementary Information:**

The online version contains supplementary material available at 10.1038/s41598-025-88082-5.

## Introduction

The American Cancer Society projects over 66,000 new cases of oral cavity, pharyngeal, and laryngeal cancers diagnosed in 2023 in the United States, and over 15,000 patients are estimated to die of this disease^1^. Squamous cell carcinoma accounts for over 95% of head and neck cancers. Apart from their shared histopathology and origin from upper aerodigestive tract mucosal sites, HNSCC are etiologically and molecularly heterogeneous and characterized by a broad spectrum of genetic aberrations^2,3^. While early-stage HNSCC can be effectively managed through curative interventions like surgery and chemoradiation, patients facing recurrence or metastasis confront a dismal prognosis, with a median survival of only 14.9 months^4^. Furthermore, a significant portion of patients remain unresponsive to chemotherapy or radiotherapy due to diverse resistance mechanisms, often enduring substantial side effects^5,6^. Consequently, HNSCC remains a substantial public health challenge both in the United States and globally.

HNSCC is characterized by diverse genetic mutations that underlie tumor initiation and progression. Key mutations encompass inactivating alterations in tumor suppressor genes like TP53, CDKN2A, and PTEN and activation of oncogenes such as EGFR, HRAS, and PIK3CA^3,7–9^. Whereas genomics has been used to aid patient stratification for targeted therapies and identify biomarkers of chemoradiation response, it has not aided in the identification of new treatment options for advanced HNSCC patients^10,11^. Furthermore, the customization of therapeutic interventions for a specific patient’s cancer is laden with assumptions and often proves inadequate. Notably, notwithstanding the detection of molecular targets, a significant proportion of malignancies exhibit inherent resistance to precision medicine strategies, with the added challenge of the rapid emergence of resistance^12,13^. Thus, there is an urgent need for improved methods to develop patient-relevant models to understand patient-to-patient variations in treatment response and eventually develop new approaches to treat head and neck cancer patients.

PDO are three-dimensional tissue models grown in vitro using tumor cells obtained directly from individual patients capable of recapitulating both the morphological and genetic attributes of the original tumor^14,15^, and predicting individual responses to chemotherapy and radiotherapy across various cancer types, including pancreatic cancer^16,17^, colorectal cancer^14,18,19^, lung cancer^20^, and others^12,21^. An advantageous feature of PDOs is their ability to faithfully reproduce the structural characteristics of in vivo tissues. This is particularly significant for organs with complex architectures, such as the head and neck regions, intestine, and liver, where cell-cell interactions and matrix constituents play pivotal roles in normal organ function^22,23^. Importantly, since PDOs are derived from a patient’s own tissue, they offer utility in understanding patient-specific differences in response to drug treatment and serve as a pre-clinical platform for identifying new therapies. This is particularly crucial in cases where tumor diversity and clonal evolution in vivo significantly impact treatment responses, like head and neck cancer.

Culture conditions for HNSCC organoids are an active area of investigation. Reported conditions include the use of media with growth factors like B27, epidermal growth factor (EGF), fibroblast growth factor (FGF), and prostaglandin E2 (PGE2), supplemented with condition media containing Wnt3a, RSPO1, RSPO3, and Noggin^24,25^. We and others have shown that the reliance on WNT signaling can vary among PDOs, implying that the specific composition of the culture medium may selectively influence both the growth and drug response of tumor organoids^26,27^. Furthermore, using Wnt agonists will impact the differentiation status of the cells and lead to the phenotypic evolution of cells during the organoid culture^27,28^. Therefore, there is interest in developing culture conditions tailored to HNSCC characteristics, effectively preserving patient tumors’ in vivo differentiation status and histological diversity. Here, we report establishing a PDO culture platform for head and neck cancer patients, offering utility for further biological investigations and serving as preclinical models to guide clinical decision-making.

## Methods and materials

### Study design

This is a pilot study designed to evaluate the feasibility of establishing PDO models suitable for radiation response assessment in patients with HNSCC at our institution, including all histologic subtypes, all stages of disease, from surgical specimens and core biopsies of metastatic lesions. Additionally, in patients where radiotherapy sensitivity could be assessed in corresponding PDOs, correlation analysis was done between in vitro radiation responses and disease control in the clinic. Informed written consent was mandatory for participant inclusion. The study protocol was approved by the Committee on Clinical Investigations (CCI), Institutional Review Board of Beth Israel Deaconess Medical Center (Boston, MA), and was conducted in accordance with the principles of the Declaration of Helsinki (IRB Protocol #: 2019P000616). Participants were enrolled from July 2020 to May 2021. Both male and female patients were recruited as sex is a biological variable.

### Sample collection

Tumor samples for PDO were collected following the determination by the surgeon and pathologist that the primary head and neck or metastatic tumor were of adequate size for research tissue sampling. The size of the research sample was at least 5 × 5 × 5 mm, up to a size deemed acceptable for research sampling without affecting diagnosis, staging, and margin examination. Research tissue was apportioned by the surgeon in a sterile manner from a bulky portion of the excised tumor so as not to affect the overall measurement of tumor size during pathologic evaluation. Sampling of tissue was avoided near resection margins, near deep aspects of the tumor, and near adjacent structures relevant for pathologic tumor staging. The surgeon transferred the sample to a sterilized container with HNSCC organoid culture medium on ice. Samples were collected by the research team for cell culture preparation within 2 h of tumor excision.

### Cell culture and media

For HNSCC PDO growth media, 2.145 mL of Reagent A, 50 µL of Reagent B, and 1.0% penicillin-streptomycin were added to 100 mL of DMEM/F-12. Components of Reagent A and Reagent B are summarized in Table 1 and Supplementary method. Primocin® (InvivoGen, Cat.: ant-pm-05) was also applied at 1:500 dilution to offer complete protection of primary cells from microbial contamination. Fresh tissues of primary tumors from patients were minced, and digested with digestion media containing collagenase/dispase, incubated with ACK lysis buffer to remove red blood cells, and then resuspended in HNSCC PDO growth media (HNTOM) for seeding. HNTOM growth media was replaced every 4 days. Detailed organoid culture procedures and recipes of digestion, resuspension, and growth media preparation were described in the supplementary method.

**Table 1:** List of supplements and growth factors used in HN PDO culture medium​.

	Supplements/growth factors	Vendor	Catalog #	Final concentration
Reagent A				
1	Bovine Pituitary Extract (BPE)	Hammond cell tech	1078-NZ	0.8 mL for 100 mL
2	B27	Thermo	17,504,001	1.0 mL for 100 mL
3	Recombinant Human FGF- Basic (FGF2)	Peprotech	AF-100- 18B	10 ng/mL
4	Recombinant Human FGF10	Peprotech	100 − 26	10 ng/mL
5	Recombinant Human EGF	Peprotech	AF-100-15	10 ng/mL
6	Recombinant Human IL6	Peprotech	200-06	100 ng/mL
7	Recombinant Human Amphiregulin	Peprotech	100-55B	50 ng/mL
8	Recombinant Human Prolactin	Peprotech	100-07	10 ng/mL
9	Human Insulin	Sigma	I2643- 250MG	10 µg/mL
10	Cholerae Toxin	Sigma	C8052-2MG	200 ng/mL
Reagent B				
1	Hydrocortisone	Sigma	1316004-200MG	0.5 µg/mL

The table specifies each reagent’s name, vendor, catalog number, and final concentration used. These reagents support the growth and differentiation of the head and neck cancer organoids in vitro, providing a conducive environment for tumor modeling.

### PDO establishment

Human tumor tissues were minced with sterile surgical scalpel (Size 21) into 0.5 to 1.0 mm fragments. ACK Lysing Buffer (ThermoFisher, Cat.: A1049201) was used to remove red blood cells in tumor tissues derived from head and neck regions, which are rich with blood vessels. Tumor fragments were then digested with digestive media for no more than 30 min. The digestion was terminated by adding resuspension media. Pellets were further digested by Accutase for 30 min and then collected by centrifugation at 1,500 rpm for 5 min. After centrifugation, supernatant was discarded, and pellets were resuspended using HNTOM growth media. The suspension was plated onto 12-well plates precoated with Matrigel. Detailed PDO establishment procedures and materials needed were described in the supplementary method.

### Whole exome sequencing

Whole-exome sequencing was performed on DNA extracted from PDOs and matched patients’ surgical tissues. Patients’ surgical tissue DNA was extracted (Qiagen 56404-QIAamp DNA formalin-fixed, paraffin-embedded (FFPE) Tissue Kit^50^) from FFPE slides with macro-dissection. Organoid DNA was extracted from cell pellets recovered from Matrigel 3D culture. Approximately 1,000,000 cells were used to perform DNA extraction for PDO DNA. Cell Recovery Solution was used to get PDO cells. DNA samples were sequenced at Novogene. Data were acquired at 12 Gb RAW data.

### Whole exome sequencing analysis

Whole exome sequencing reads were aligned to human genome version hg19 using BWA (v.0.7.8-r455). Alignment, “.bam” files were sorted with Samtools (v1.0), and duplicate reads were removed with Picard (v1.111, http://broadinstitute.github.io/picard)^29,30^. Candidate SNV variants were called using GATK (v3.8). ANNOVAR (v2015Dec14) was used to annotate population-level allele frequencies across the 1000 Genomes, Exome Aggregation Consortium (ExAC), and National Heart, Lung, and Blood Institute (NHLBI) Exome Sequencing Project (ESP) germline variant consortia^31^. To enrich variant calls for somatic mutations, SNVs with allele frequencies > 1E-5 in at least one of the three germ-line variant consortia were removed. Mutational consequence and amino acid changes of SNVs in transcript coding regions were annotated using the VariantAnnotation (v1.40.0) R package^32^. Additionally, SNVs were characterized according to their presence in the COSMIC database in HNSCC or any other cancer type (https://cancer.sanger.ac.uk/cosmic/download, downloaded on March 21, 2023)^33^. Copy number variant calling was performed using CNVkit (v0.9.9). Copy number amplifications and deletions were called across autosomal chromosomes using copy number estimate thresholds, greater than 2.5 and less than 1.5, respectively^34^.

For each variant category, the global concordance of variant profiles between samples was assessed based on the similarity of their shared calls. For SNVs, similarity between variant call profiles was measured using Jaccard Similarity, measured as the intersection of variant calls relative to the union between two samples. For CNVs, similarity of overlapping amplified and deleted regions was measured using Szymkiewicz-Simpson similarity, measured as the fraction of overlapping variant regions between two profiles relative to the smaller set of variant regions using the CNVMetrics (v1.1.0) R package.

Variant calls, shared across subject-wise PDO and FFPE sample pairs, were further annotated based on membership of functional categories, including genes commonly mutated in HNSCC, known tumor suppressors and oncogenes based on “Cosmic Census Genes” (https://cancer.sanger.ac.uk/census, downloaded on Jan 12, 2023), and “hallmarks” of cancer gene sets from the mSigDB database (https://www.gsea-msigdb.org/gsea/msigdb/human/collections.jsp#H, v7.5.1)^33,35–37^. Specifically, mSigDB functional categories assessed were divided into three categories: epithelial-mesenchymal transition (EMT), Proliferation, and DNA Repair. Proliferation genes represented the aggregate of six hallmark gene sets: G2/M checkpoints, E2F transcription factor targets, Myc targets versions 1 and 2, p53 pathway, and mitotic spindle assembly. DNA repair genes represented the aggregate of three hallmark gene sets: DNA repair, and genes up- and down-regulated in response to ultraviolet radiation.

### Morphological and histological analysis

PDOs were plated at a density of 25,000 cells/mL, and images were taken every day for 12 days.

For preparation of histologic slides, PDOs were grown in chamber slides. At around 70% confluency, PDOs embedded in Matrigel in the chamber were fixed in 4% PFA at room temperature for 2 h. The PDOs were then incubated with hematoxylin solution for 10 min, washed twice with PBS, and then scraped and sandwiched between two layers of Histogel (Thermo Scientific™, Cat. #HG-4000-012) using a cryomold (Tissue-tek Cryomold Standard Cat. #4557). The sandwich was then transferred to a tissue cassette and fixed in 10% formalin (Sigma Aldrich, Cat. #HT501128). Histologic processing and hematoxylin and eosin (H&E) staining were performed per routine by the institutional research histology core facility.

Antibodies used for fluorescent immunostaining and their antibody catalog numbers were listed below: P16 (BD Biosciences, Cat. #551154), Cytokeratin 10/13 (DE-K13) (Santa Cruz Biotechnology, Cat. #sc-6258), Cytokeratin 5 (RCK103) (Santa Cruz Biotechnology, Cat. #sc-32721), HCAM/CD44 (DF1485) (Santa Cruz Biotechnology, Cat. #sc-7297), DAPI (Thermo Scientific™, Cat. #62248).

### Immunofluorescence staining

Slides were incubated with blocking buffer (5% BSA/0.5% Tween 20 in PBS) for 1 h at room temperature in a humidified chamber. Primary antibodies were diluted 1:1000 in blocking buffer. After removing blocking buffer from sections, primary antibody was added to cover each section. Slides with primary antibody added were incubated overnight at 4℃ in a humidified chamber. On day 2, secondary antibody was added at room temperature for a 1-hour incubation. Slides were washed with PBS twice and stained with 1:100-diluted DAPI reagent (Thermo Scientific™, Cat. #62248) for 5 min and washed with PBS. Slides were finally mounted with ProLong Gold Antifade Reagent (Thermo Scientific™, Cat. #62248), sealed with nail polish, and imaging was conducted followed by long-term storage at −20℃.

### Radiation assay

Four days before applying radiation treatment, 3000 ~ 5000 cells/well were seeded into 96-well plates (Corning, Cat. #3904) and maintained in a 37℃ incubator. Culture media were refreshed right before applying radiation treatments. Five doses of radiation (0, 2, 4, 6, 8 Gy) were applied to the cells using X-Rad 320 Biological Irradiator platform (PRECISION X-ray, North Branford, CT) at 320 KV and 12.5 mA with the guidance of a radiation oncologist. Six days after radiation, cells were harvested, and cell viability was assessed using the CellTiter-Glo® 3D Cell Viability Assay (Promega, Cat. # G9681) per manufacturer’s protocol.

### Chemodrug assay

One day before applying chemodrug treatment, 3000 ~ 5000 cells/well were seeded into 96-well plates (ThermoFisher, Cat. #Nunc 167008) and maintained in a 37℃ incubator. Seven concentrations (0, 0.008, 0.04, 0.2, 1, 5, 25 µM) of either Cisplatin or Carboplatin were added to the cells using Tecan D300e digital dispenser (Tecan Life Sciences, Switzerland). Three days after drug treatment, cells were harvested, and cell viability was assessed using the CellTiter-Glo^®^ 3D Cell Viability Assay (Promega, Cat. # G9681) per manufacturer’s protocol. PDO cell viability after drug treatment was normalized to the mean number of untreated cells. AUC-based response to drug concentrations was analyzed using dose-response data analysis, “drda,” R-package (https://cran.r-project.org/web/packages/drda/index.html)^38^.

## Results

### Developing WNT-free media conditions for the culture of head and neck tumor organoids

To achieve WNT-free conditions, we expanded the conditions we recently established for breast cancer organoids^39^due to the shared ectodermal lineage during the development of breast and head and neck tissues. For instance, bovine pituitary extract (BPE) is an effective serum-free supplement for epithelial cells. When combined with insulin, EGF, and hydrocortisone, the factors promote the growth of epithelial cells while preventing excessive fibroblastic growth^40^. Additionally, interleukin-6 (IL-6) is a critical component of the cancer microenvironment, which can be secreted by cancer cells or their stromal counterparts, such as cancer-associated fibroblasts (CAFs). Elevated levels of IL-6 have been reported in the serum and other biological fluids of HNSCC patients^41^. Studies have also demonstrated that IL-6 stimulates the proliferation of small intestinal organoids through a STAT3-mediated signaling pathway^42^, which is relevant in head and neck cancer as dysregulated STAT3 signaling in HNSCC contributes to malignant behaviors, including enhanced growth, survival, and resistance to therapies^43^. The tumor microenvironment has been identified as one of the variables contributing to the growth and invasion of HNSCC. Constantly active CAFs, found inside or close to the tumor mass, have been linked to having a solid HNSCC modifying impact and playing a significant role in areas such as drug resistance^44^. To minimize fibroblast overgrowth during the establishment of tumor organoids, we include cholera toxin, which is known to exert a growth inhibitory effect on fibroblasts^45^. EGFR and FGF2 are highly expressed in up to 60–90% of HNSCC tumors^46,47^, and Marianne et al. revealed that frequent coexpression of FGF2 and FGFRs in HNSCC cell lines, thereby instituting an autocrine loop that, alone or in collaboration with EGFR, drives cell growth^46^. Also, FGF10 has been proven to support the establishment and maintenance of the HNSCC organoid cultures^24^. Prolactin receptor and amphiregulin expression is elevated in HNSCC and is associated with poor clinical outcomes, identifying a need for the inclusion of prolactin and amphiregulin in the culture media^48,49^. Taking these factors into account, we developed a media, as outlined in the methods section, to establish and maintain head and neck tumor organoids.

### Patient-derived organoids recapitulate the morphological characteristics of patient’s tumor

**Fig. 1:**
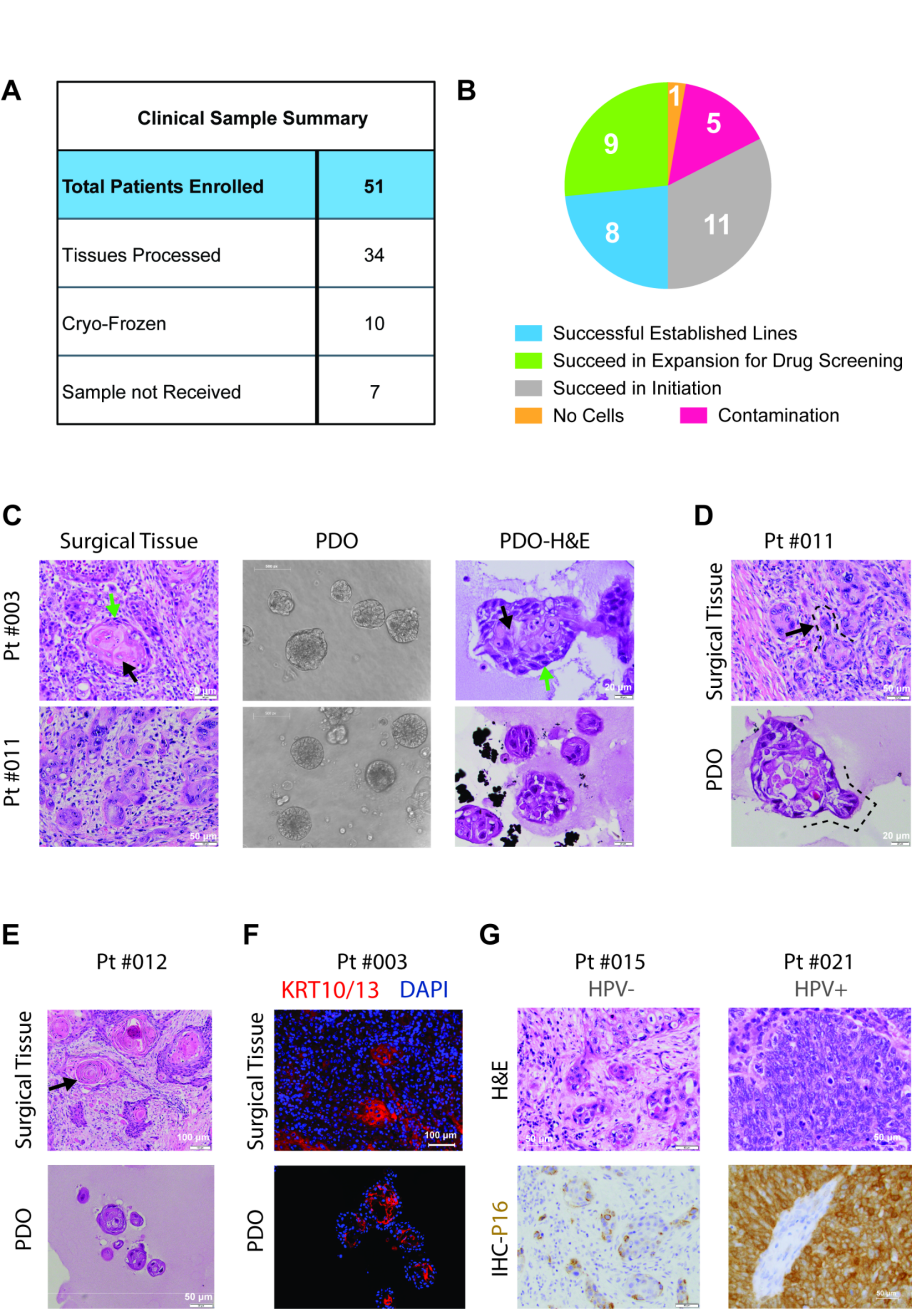
Morphological and pathological recapitulation of patient-derived organoids with matched tumor sources. (**A**) Summary of clinical sample acquisition and subsequent PDO establishment. (**B**) Pie chart illustrating the categories of PDO establishment status among stages. (**C**) H&E staining of matched surgical tissue and PDOs for Pt #003 and Pt #011. (**D**) Pathological feature of elongated pseudopodia in matched surgical tissue and PDOs for Pt #011. (**E**) Pathological feature of keratin pearl in matched surgical tissue and PDOs for Pt #012. (**F**) KRT 10/13 IF staining in matched surgical tissue and PDOs for Pt #003. (**G**) IHC staining for p16 as a surrogate marker for HPV status diagnosis for HNSCC patients (Pt #015 and Pt #021).

In this study, we recruited a cohort of 51 patients, and collected tumor tissues for processing from 34 cases (Fig. 1A, Supplementary Table S1). Of the 34, one tissue did not yield any tumor cells, and five were lost to contamination. Among the remaining 28 samples, eleven samples initiated tumor organoid growth by forming organoids; however, it ceased within 1–2 passages (Fig. 1B) and nine samples succeeded in initiation and progressed to expansion for 4–5 passages, generating enough cells for radiation and drug screen assays but did not sustain multiple passages to yield stable lines. The remaining eight samples expanded well beyond five passages and were successfully established as organoid lines for further experimentation (Fig. 1B). Overall, we achieved a 50% (17/34) success rate in establishing the PDO culture that can be expanded for 4–5 passages. Those cultures that yielded no tumor cells or contaminated or had tumor cells but ceased growth within 1–2 passages were considered failures.

Patient characteristics of the PDO lines pursued for further experiments are shown in Supplementary Table S2. Microscopic evaluation showed that PDOs exhibited similar histopathologic features to the epithelial component of the matched surgically resected squamous cell carcinoma from the patient. For instance, the conventional well-differentiated keratinizing squamous cell carcinoma resected from pt #003 comprised variably sized tumor cell nests with heterogeneous cytomorphology (Fig. 1C, upper left). In the surgical resection sample, the periphery of these tumor nests was enriched for immature-appearing “basaloid” cells with scant cytoplasm and dark, ovoid nuclei (resembling the proliferative basal layer of normal stratified squamous epithelium; green arrow). In contrast, cells near the center of these tumor nests were larger, with abundant eosinophilic cytoplasm (resembling the more mature, superficial cells of the stratified squamous epithelium; black arrow). Both basaloid and mature-appearing cells with abundant eosinophilic cytoplasm comprised the corresponding PDOs (Fig. 1C, upper right). For the moderately differentiated squamous cell carcinoma from pt #011, the degree of nuclear pleomorphism in the PDO appeared similar to that of the corresponding surgical tissue (Fig. 1C, lower panel). The PDO for pt #011 (HN-011) also exhibited elongated pseudopodia or bud-like extensions of tumor cells (highlighted by dashed lines), mirroring the growth pattern observed in the surgical tissue (Fig. 1D).

“Keratin pearls” (KP) are distinctive histologic structures in squamous cell carcinomas, typically in well- to moderately-differentiated keratinizing tumors. KP are composed of concentrically arranged rings of squamous cells with increasing degrees of keratinization (manifesting as abundant, eosinophilic cytoplasm) towards the center of the tumor cluster. KP-like structures were observed in HN-012 derived from well- to moderately-differentiated squamous cell carcinomas (Fig. 1E, black arrows), identical to the matched surgical tissue histology.

Cytokeratins (KRTs) serve as valuable markers for characterizing tissue differentiation, a feature directly applicable to the study of malignant tumors^50,51^. For instance, certain squamous cell carcinomas exhibit elevated expression levels of cytokeratins 10/13^50,51^, whereas KRT18 is mostly expressed in adenocarcinomas^52^. In pt #003, KRT10 and KRT13 were detected within the central cells of the organoid, consistent with expression in the matched patient tumor tissue section (Fig. 1F).

Strong, diffuse nuclear and cytoplasmic immunohistochemical expression of p16 by tumor cells has been regarded as a surrogate biomarker for high-risk HPV in squamous cell carcinomas of the uterine cervix and oropharynx^53,54^. Among the lines we established, HN-021 was derived from an HPV + tumor, and the remainder was considered HPV-negative based on either the absence of p16 staining (HN-015) or the non-oropharyngeal primary site of the tumor (pt #011, 012, and 029) (Fig. 1G; Table 2). This indicates our culture condition can establish and maintain PDOs from both HPV-negative and -positive tumors in HNSCC patients, highlighting the capability of this platform as a promising pre-clinical model.

**Table 2:** Clinical characteristics of head and neck cancer patients from whom tumor tissue samples were collected for organoid establishment and experimentation.

Organoid ID	Age (y)/Sex	Tumor site	Stage group	Primary tumor stage	Nodal stage	Radiation therapy dose (Gy)	Chemotherapy	Follow-up
HN-003	83/M	Oral cavity (floor of mouth)	IVA	T2	N2b	60	None	Died 19 months post-treatment (cause unknown)
HN-011	62/M	Larynx	IVA	T4a	N0	60	Cisplatin	Alive; NED 34 months post-treatment
HN-015	52/M	Larynx	IVB	T4a	N3b	60	Carboplatin	Died 15 months post-treatment (metastasis to lung versus new primary lung squamous cell carcinoma)
HN-018	88/M	Skin (cheek)	II	ryT3	N0	44	None	Died 4 months after surgery with local recurrence
HN-029	78/F	Oral cavity (buccal)	IVB	T4b	N0	60	Carboplatin	Alive; NED 24 months post-treatment

For each patient, the table provides their age, sex, tumor site, cancer stage, radiation dose received, chemotherapy type, and treatment outcome with follow-up duration post-treatment.

### Cell biological features of PDO models

**Fig. 2:**
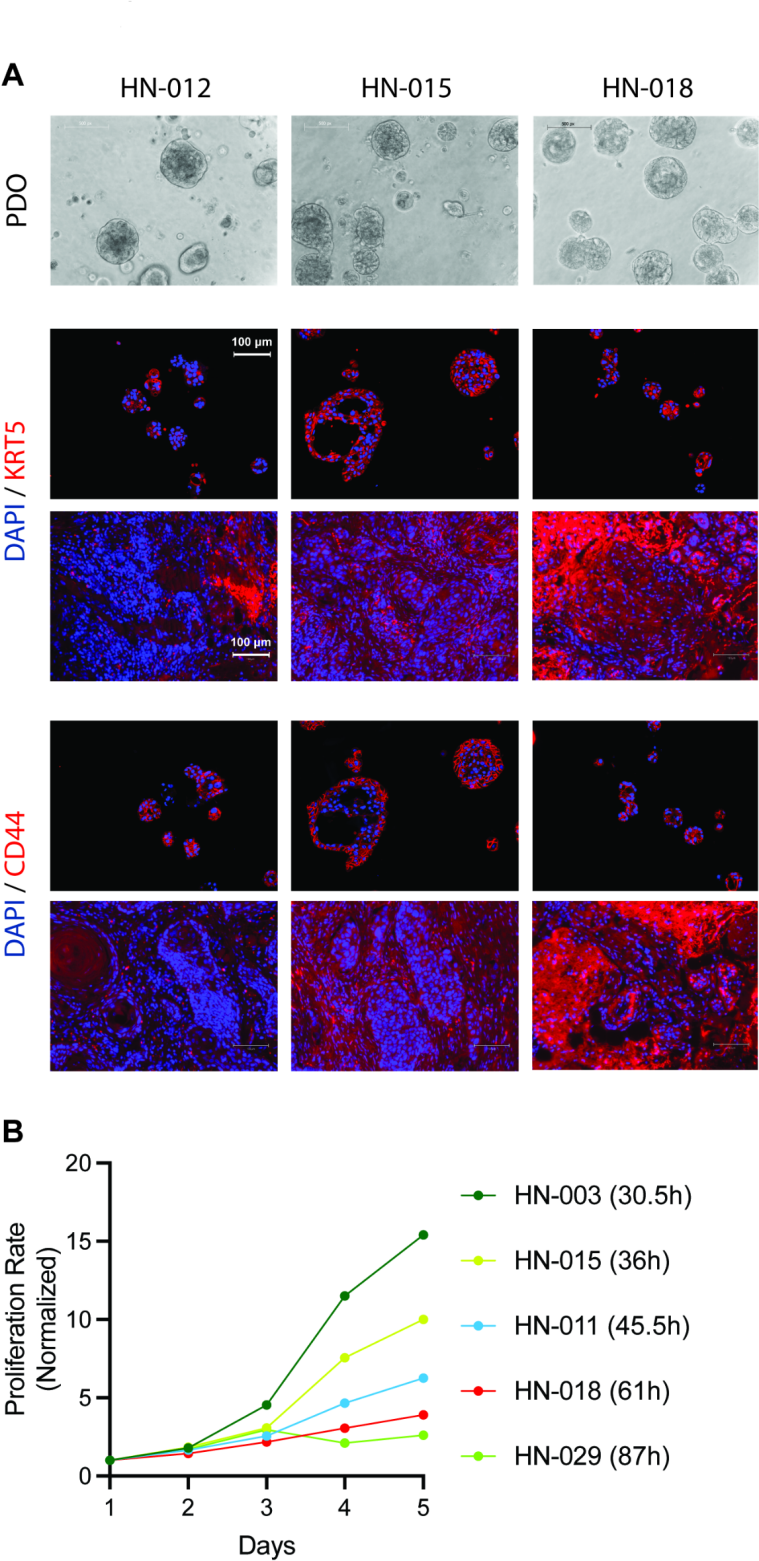
Cell biological features of PDO models. (**A**) KRT 5 and CD44 IF staining in matched surgical tissue and PDOs for Pt #012, Pt #015, Pt #018. (**B**) Proliferation rate of five HNSCC PDO lines with cell doubling time ranging from 30.5 to 87 h.

To understand the cell biological properties of the organoid cultures, we immunostained for cell membrane proteins and cytokeratins. CD44/HCAM (homing cell adhesion molecule) expression is associated with tumor growth and metastasis and is a potential marker for cancer stemness and poor prognosis in HNSCC^55,56^. HCAM is a hyaluronic acid receptor and interacts with ligands such as osteopontin^57^. This involvement allows HCAM to mediate cell-cell and cell-matrix interactions, which are critical in cell adhesion and migration. As Fig. 2A shows, all three patient-derived organoid lines showed CD44/HCAM staining, mainly expressed at the cell membrane. Also, KRT5, regarded as a proliferation marker^24^, was detected in all three PDO lines (Fig. 2A). The cell doubling times of five PDO lines ranged from 30 to 87 h (Fig. 2B), demonstrating that our culture media is not overly supplemented with growth factors to normalize the proliferation rates of organoid lines from different patients.

### Genetic alterations were shared between PDOs and patient tissues

**Fig. 3:**
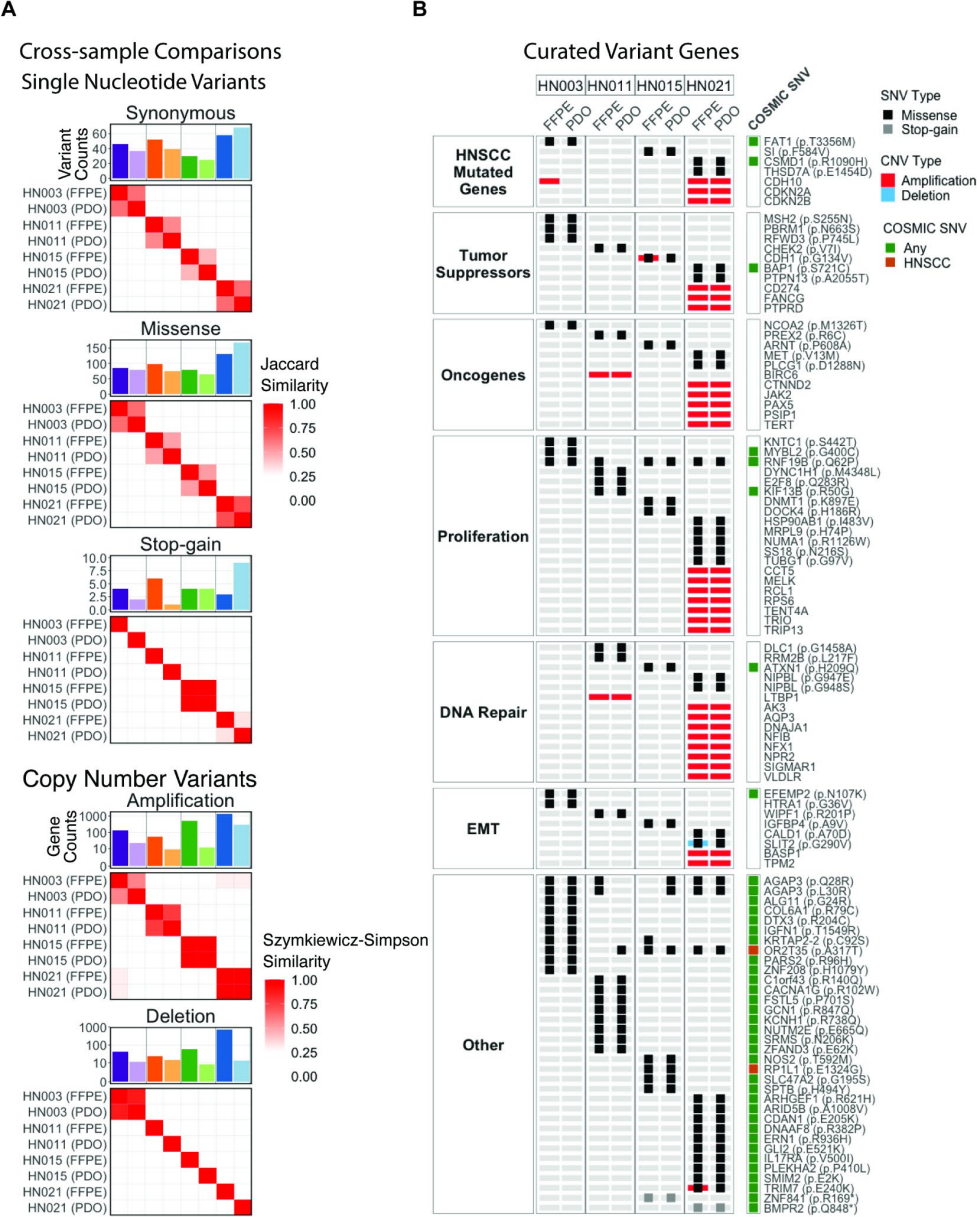
Genetic concordance between PDOs and surgical tissue FFPEs revealed by whole exome sequencing. (**A**) Single nucleotide variants (SNV) and Copy number variant (CNV) calls and similarity between matched PDO and FFPE pairs. For SNV calls, Jaccard similarity is based on individual variant calls. For CNV calls, Szymkeiwicz-Simpson similarity is reported based on genomic regions of copy number variation. (**B**) Evaluation of potential major genetic mutations that replicate between matched FFPE and PDO samples.

To understand the genomic fidelity of PDOs, we performed exome sequencing on four pairs of PDOs with matched surgical specimens obtained from FFPE samples. Single nucleotide variants (SNV) and Copy number variant (CNV) calls between matched PDO and FFPE pairs were analyzed for their global similarity and shared calls for HNSCC-relevant variants. SNVs including synonymous (25–68 per sample), missense (65–167 per sample), and stop-gain variants with variant calls (1–9 per sample), and CNV calling identified amplified and deleted regions comprising genes per sample ranging from 9−1,334 and 8–710, respectively (Fig. 3A, Supplementary Table S3). For CNVs, we observed consistently greater variant region calls in FFPE samples compared to their respective matched PDO samples. However, this trend was not observed for SNV calls. For all variant categories, similarity measures yielded higher levels of similarity between matched samples compared to minimal similarity between unmatched samples (Fig. 3A, Supplementary Table S4).

To evaluate potential major genetic mutations that replicate between FFPE and PDO samples, we explored shared variant calls between matched samples in several gene categories related to HNSCC malignancy, as well as variant calls reported in the COSMIC database^33^ (Fig. 3B). This revealed 70 replicated missense and amplification variant calls across commonly mutated HNSCC genes^35^, tumor suppressors, oncogenes, genes involved in proliferation, DNA repair, and EMT. Moreover, we identified an additional 34 replicated missense and stop-gain calls that have been previously reported to the COSMIC database^33^, including two missense SNVs, OR2T35 (p.A317T) and RP1L1 (p.E1324G), in HNSCC patients. There are also exceptions that show distinct profiles between PDO and surgical tumor tissue. For example, amplifications of CDH10 and CDH1 are detected in surgical tissues but not in HN-003 and HN-015, respectively. Moreover, deletions of SLIT2 are detected in surgical tissue but not in HN-021. Conversely, missense mutation of OR2T35 is only detected in PDO but not in surgical tumor tissue (Fig. 3B). In addition to technical factors affecting the accuracy of variant calling from whole exome sequencing, such discrepancies could arise from intratumoral heterogeneity, as different parts of the tumor are used for pathology and organoid generation, and this could result in differences of mutational profiles between surgical tissue and PDO.

### Radiation and chemodrug testing for in vitro assessment of patient responses

**Fig. 4:**
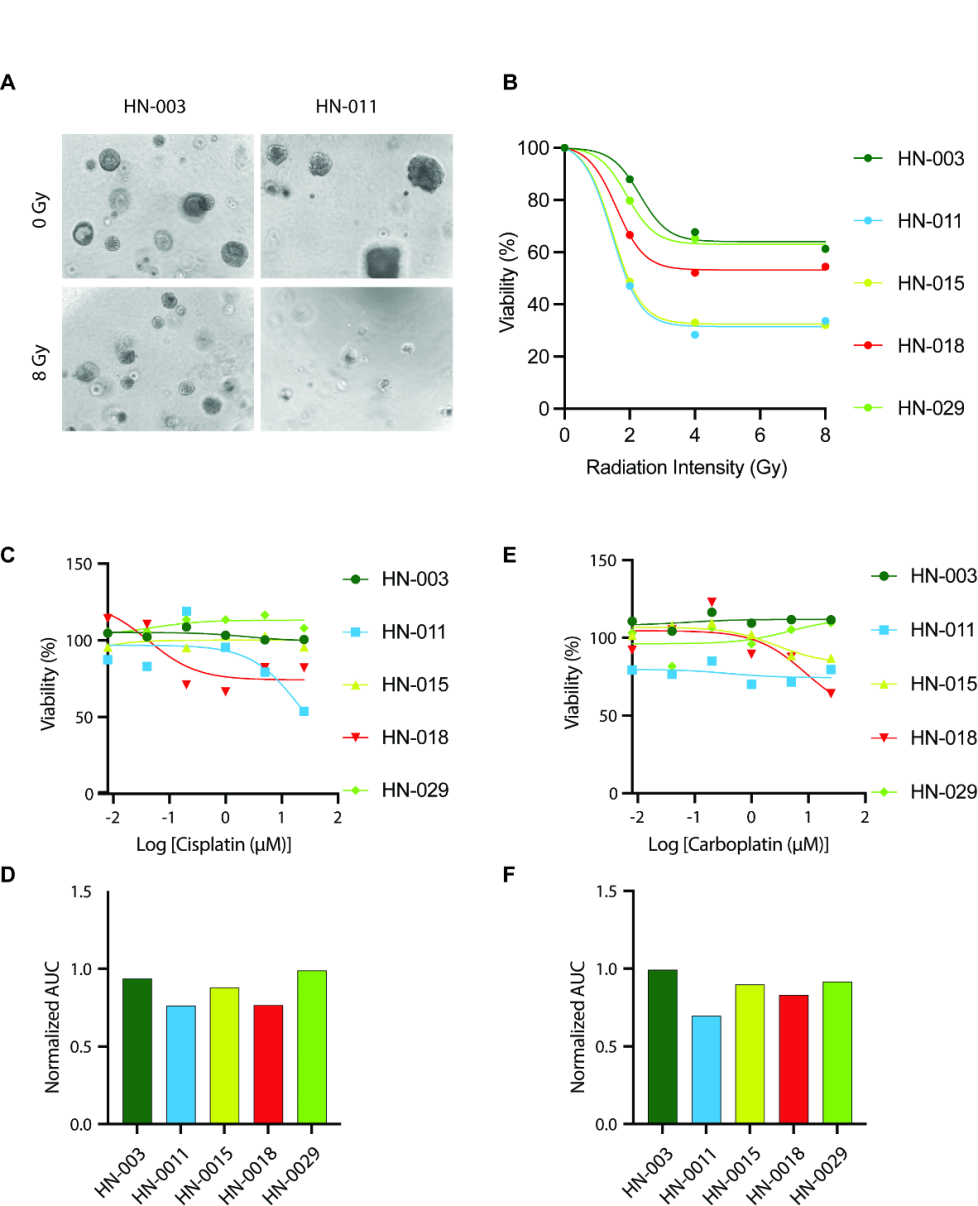
Radiation and chemodrug testing for in vitro assessment of patient responses. (**A**) Representative images of HNSCC PDOs (HN-003 and HN-011) six days after radiation treatment. (**B**) Radiation responses of five PDO lines tested under 0, 2, 4, 6, 8 Gy doses. (**C**,**E**) Chemodrug responses of five PDO lines tested under Cisplatin and Carboplatin. (**D**,**F**) Normalized Area under Curve (AUC) values for Chemodrug responses to Cisplatin and Carboplatin.

To understand if PDOs can be used to model patient’s radiation sensitivity, PDOs were exposed to increasing doses of radiation treatment. As shown in Fig. 4A, HN-003 PDO did not show any significant change in organoid size and number, whereas HN-011 showed a dramatic decrease in organoid survival in response to 8 Gy of radiation, demonstrating the ability to use organoid viability as a metric to assess radio-responsiveness. Organoid lines were subject to increasing doses of radiation, and cell viability was measured six days later. HN-011 and HN-015 PDOs were the most sensitive among the five lines tested (Fig. 4B). The same five PDO lines were also analyzed for sensitivity to cisplatin and carboplatin, two drugs commonly used in combination with radiation to treat patients with HNSCC. Cell viability was assessed after 3 days of treatment. The five lines exhibited differential yet low responsiveness to both cisplatin and carboplatin treatment (Fig. 4C,E). Among the lines tested, HN-011 showed the highest sensitivities to both cisplatin and carboplatin as determined by the normalized area under the curve (AUC) measurements (Fig. 4D,F).

Interestingly, pt #011, from whom the HN-011 PDO line was generated, received 60 Gy radiation and cisplatin for stage IVA larynx cancer; he is alive with no evidence of disease recurrence as of October 2023, 34 months after completion of treatment (Table 2), suggesting the ability of PDOs to predict clinical sensitivity. Despite the observed sensitivity of HN-015 to radiation, pt #015 received 60 Gy radiation and carboplatin for stage IVB larynx cancer but died 15 months after completion of treatment following the development of squamous cell carcinoma in the lung. HN-015 cells had the fastest doubling time among all the lines (Fig. 2B); whether this contributed to mortality is unclear. Furthermore, it is also unclear whether the lung lesion was a metastasis from the patient’s laryngeal cancer or a separate primary tumor of the lung. Pt #003 received 60 Gy radiation for stage IVA oral cavity cancer and died 19 months after treatment (Table 2). Although it is unclear whether the death was due to cancer recurrence, the lack of response to radiation treatment is consistent with the HN-003 PDO from pt #003, showing evidence of radioresistance (Fig. 4B). Pt #018 received palliative radiation preoperatively for recurrent cutaneous squamous cell carcinoma. The tumor recurred three months after surgery, and the patient died a month later. Consistent with the clinical outcome, HN-018 showed radio-resistant behavior to all the doses of radiation tested. Pt #029 received 60 Gy radiation and carboplatin for stage IVB buccal squamous cell carcinoma; she is alive with no evidence of disease 24 months after completion of treatment. Interestingly, the PDOs from this patient were radio- and chemo-resistant. The factors contributing to the lack of correlation between clinical and in vitro responsiveness are unclear.

Although a larger cohort of matched response measurements is needed to understand if PDOs can be effectively used to personalize treatment options for HNSCC patients, our data identifies promise for the ability to use PDOs to assess radio- and chemotherapy responsiveness.

## Discussion

Here, we report the development of culture conditions for the establishment of organoids from HNSCC tumors under WNT-free media conditions. By comparing histological, cell biological, genomic, and chemoradiation responses between PDO and matched patients, we demonstrate that organoid conditions are not only effective in retaining the molecular properties of the patient tumor but also effective in modeling treatment responses of the patient. Together, we believe that our HNSCC PDO platform reported here is poised to enhance our understanding of HNSCC pathogenesis, create opportunities for developing personalized treatment options, and create patient-derived models for understanding chemotherapy and radiation responsiveness and resistance.

Our unique culture media offers advantages and improvements. Published studies have developed several methods for HNSCC PDOs culture, with major procedures being largely similar^24,25,58–61^. However, unlike most culture media, which incorporate Wnt3a or CHIR99021 to activate Wnt pathway^25,59–63^, our approach avoids these components purposely (see Supplementary Table S5 for comparison with previously published recipes). Although WNT agonists are added in PDO culture media for gastrointestinal cancers, such as colorectal cancer that have Wnt activation in > 90% of cancers^64–66^, HNSCCs exhibit less pronounced Wnt activitation. By excluding Wnt agonists, our Wnt-free media minimizes the risk of altering the differentiation status of cells or driving phenotypic evolution during culture and reduces the prevalence of stem cell-like phenotypes^27,28^. By accommodating Wnt-independent cell populations, this method also preserves intratumoral heterogeneity, allowing for a more comprehensive representation of the bulk tumor. Moreover, the absence of Wnt signaling creates an opportunity to investigate alternative growth and survival pathways, potentially uncovering mechanisms of resistance to Wnt-targeted therapies^66^. We believe that the design of WNT-free media conditions is the key advantage of our HNSCC PDO culture media.

The criteria for defining a successful PDO establishment differ across studies. In our work, we adopted a more rigorous standard, defining successful PDOs as those capable of being passaged at least four passages, with stable lines requiring sustained viability beyond five passages. Using this higher threshold for success, our culture medium achieved a success rate exceeding 40% (14 out of 34 samples), demonstrating its effectiveness for both basic research and clinical applications. Pairwise comparison of PDOs with corresponding patient tissues and clinical response identifies concordance both in molecular features and clinical outcomes.

Given the intricate nature of head and neck cancers, effective and reliable implementation of a multimodal therapeutic strategy necessitates the consideration of numerous factors^67^. However, current treatment strategies, incorporating surgery, chemotherapy, and radiotherapy, often exert substantial toxicity on patients. Since HPV-positive HNSCC has a more favorable prognosis^4,7,68^, strategies for treatment de-intensification for HPV-positive patients are urgently warranted to reduce treatment-related toxicity. Preclinical models like PDX (patient-derived xenograft) have been regarded as ideal for personalized medicine^69–71^. There is a clear demand for an in vitro PDO model capable of stratifying patients using validated biomarkers and treatment efficacy assays. Whereas cell lines have been the workhorse for all preclinical studies for decades, the PDO model provides a significant advantage. PDOs are more patient-relevant, they do not suffer the significant culture-induced genetic and epigenetic drifts associated with cell lines; and they retain a greater level of patient-relevant tumor heterogeneity compared to established cell lines. Furthermore. our group also found that there’s a close correlation between PDO and PDX in drug response and genetic background^27^. Thus, it is feasible to use in vitro PDO model to identify treatment combinations that increase efficacy and exclude those that are likely to be unresponsive, which may further increase the probability of a positive clinical response while reducing the burdens of toxicity and treatment costs associated with HNSCC management.

While these results are promising, our study has limitations. Firstly, despite observed concordance between PDO responses and clinical outcomes, the small sample size limits the statistical power to draw definitive conclusions about the predictive accuracy of PDO responsiveness for patient clinical outcomes for HNSCC. Secondly, PDOs may not entirely capture the complexity and heterogeneity of the original tumor, potentially affecting the accuracy of drug response predictions. Furthermore, organoids primarily represent the tumor’s epithelial component and may not fully replicate the tumor microenvironment, including stromal cells, immune cells, and vasculature, which play pivotal roles in tumor growth and therapy response, resulting in limited representativeness. Additionally, the time required to establish and expand organoids, often several weeks, may not be compatible with the urgent clinical scenarios or immediate treatment decisions some patients require. Despite these limitations, PDOs offer significant potential for discovery research.

Presently, the repertoire of FDA-approved targeted therapies for head and neck patients is limited, with EGFR and immune checkpoint inhibitors such as pembrolizumab and nivolumab among the few^2,72^. Expanding our biobank to encompass patients demonstrating varying responses, including sensitivity and intrinsic resistance, will facilitate establishing in vitro models. These models will enable the investigation of potential mechanisms underlying chemoradiation resistance, including further establishment of lines with acquired resistance, which could be aimed at identifying therapeutic targets that can sensitize patients to current treatment options.

HNSCC frequently exhibits a strong immunosuppressive environment, typically characterized by a scarcity of T cells within the tumor microenvironment^73^. The utilization of adoptive cell therapy involving T cells engineered with tumor-specific T-cell receptors (TCRs), capable of recognizing unique cancer-related neoantigens, represents a promising approach to address the current limitations of available treatments. Our group has previously established a co-culture platform, facilitating the expansion of tumor-targeting T cells from peripheral blood samples obtained from patients. This platform holds the potential to be further harnessed for the investigation of T-cell-based immunotherapy approaches tailored to head and neck cancer patients^74^.

In summary, these advantages and promising applications highlight the potential of Wnt-free media in enhancing physiological relevance and experimental utility of HNSCC organoid models. While further validation is necessary to fully confirm these benefits, we believe that the PDO platform reported here has the potential to not only enhance discovery studies but also create opportunities for the development of strategies for personalized medicine.

## Electronic supplementary material

Below is the link to the electronic supplementary material.

Supplementary Material 1

Supplementary Material 2

## Data Availability

All authors will follow the FAIR principles (Findability, Accessibility, Interoperability, Reproducibility) for data access as a condition of publication. Sequence data that support the findings of this study have been deposited in the database of Genotypes and Phenotypes (dbGaP) with the primary accession code phs003755.v1.p1. All relevant data are available from the corresponding author on request (Senthil K. Muthuswamy: senthil.muthuswamy@nih.gov).
