# Supplementary material for: Head and neck tumor organoid grown under simplified media conditions model tumor biology and chemoradiation responses
Source: Sci Rep. 2025 Jul 7;15:24221. doi: 10.1038/s41598-025-88082-5 (PMC12234821; doi:10.1038/s41598-025-88082-5)
Supplement: Supplementary file 1 — Supplementary Material 1 [file 41598_2025_88082_MOESM1_ESM.pdf]

Method for Generation and Establishment of Organoids from Head & Neck Cancer Patient Tumor Specimens

This protocol outlines the principal steps and methodologies used to generate tumor organoids in our laboratory. Although the principles outlined here are shared with the protocol we use for any organ, the media and some conditions outlined here are optimized for generating Patient-Derived tumor Organoid (PDO) cultures directly from surgical resections of human HNSCC tumors. The initial organoid culture that develops after plating the processed tissue is referred to as Passage 0 (P0); all subsequent passages should be kept track of and serially numbered.

Terminologies used to categorize the success or failures of organoid cultures:

1) *Established organoid cultures*: Cultures that are initiated by forming organoids and sustained for more than 5 passages are referred to as stable lines for future experimentation.
2) *Initiated organoid cultures:* Cultures that succeeded in initiation and progressed to expand for 4-5 passages, generating enough cells for radiation and drug screen assays but were not viable after 5 passages.
3) *Initiated but failed to expand cultures:* Cultures that initiate as organoids but do not survive past 1-2 passages.
4) *Failed organoid cultures:* Cultures that yielded no tumor cells, or contaminated are considered failures.

Materials Needed

Materials	Vender	Catalog #
Collagenase/Dispase (100 mg/mL)	Sigma	11097113001
Accutase® solution	Sigma	A6964
DMEM, high glucose	ThermoFisher	11965092
DMEM/F-12, HEPES	ThermoFisher	11330032
Y-27632 dihydrochloride	Tocris	1254
Matrigel® Growth Factor Reduced (GFR) Basement Membrane Matrix, LDEV-free	Corning	354230
Penicillin-Strepomycin (10,000 U/mL)	Gibco	15140-122
Bovine Serum Albumin (Heat shock fraction)	Sigma	A7906
EASY strainer (100 µm)	Greiner Bio-one	542000
4-well chamber slides	Corning	354114
CryoStor CS10 Cell Freezing Medium	STEMCELL technologies	07930
Primocin®	InvivoGen	ant-pm-05
ACK lysis buffer	ThermoFisher	A1049201

Head & Neck Tumor Organoid Media (HNTOM) Recipe

	**Supplements/Growth factors**	**Vendor**	**Catalog #**	**Final concentration**
Reagent A				
1	Bovine Pituitary Extract (BPE)	Hammond cell tech	1078-NZ	0.8 mL for 100 mL
2	B27	Thermo	17504001	1.0 mL for 100 mL
3	Recombinant Human FGF- Basic (FGF2)	Peprotech	AF-100- 18B	10 ng/mL
4	Recombinant Human FGF10	Peprotech	100-26	10 ng/mL
5	Recombinant Human EGF	Peprotech	AF-100-15	10 ng/mL
6	Recombinant Human IL6	Peprotech	200-06	100 ng/mL
7	Recombinant Human Amphiregulin	Peprotech	100-55B	50 ng/mL
8	Recombinant Human Prolactin	Peprotech	100-07	10 ng/mL
9	Human Insulin	Sigma	I2643- 250MG	10 µg/mL
10	Cholerae Toxin	Sigma	C8052-2MG	200 ng/mL
Reagent B				
1	Hydrocortisone	Sigma	1316004-200MG	0.5 µg/mL

Media Components & Recipes:

Digestion Media:

DMEM, high glucose

1:100 dilution of 100 mg/mL stock Collagenase/Dispase

1.0% Penicillin-streptomycin

Preparation Tip:

- Prepare only the needed amount, fresh just before use, and discard the unused portion of the media.

Resuspension Media:

DMEM, high glucose

1.0% Penicillin-streptomycin

1.0% BSA

Preparation Tip:

- Add 1% BSA by weight to DMEM+1.0% Penicillin-streptomycin containing media and stir to dissolve the BSA. Once dissolved, filter sterilize using 0.2 μm filter and store at 4℃.

HNTOM Growth Media:

DMEM/F12: 100 mL

2.145 mL of HNTOM Growth factors cocktail (Reagent A)

1.0 % Penicillin-streptomycin

50 μL Hydrocortizone (1.0 mg/mL) (Reagent B)

Preparation Tip:

- Do NOT mix Reagent A and Reagent B by themselves as the alcohol in Reagent B solution will denature the growth factors in Reagent A.
- After adding all ingredients filter the media through a 0.2 μm filter.
- Store prepared media at 4℃ for NO more than one month.

Culture Media:

HNTOM growth media

5.0% Matrigel® Growth Factor Reduced (GFR)

10 µM Y267632

1:500 dilution of Primocin stock (50 mg/mL)

Preparation Tip:

- Prepare fresh. You may premix HNTOM growth media and ROCK inhibitor and keep on ice.
- Add Matrigel just before use. Discard unused portion of the media.
- Rock inhibitor stock (10 mM) is prepared in sterile Phosphate Buffered Saline.

Freezing Media:

Cryostor CS10 Cell Freezing Medium

PROCEDURE FOR PROCESSING TISSUES

1. Place the tumor tissue received from the surgery in a 35 mm petri dish. You may choose to take a small section of the tissue and fix it for histology processing. Another piece of the tissue can be snap-frozen in liquid nitrogen for future use to isolate DNA/RNA. These are optional and will depend on the amount of surgical material received.

2. Add 1.0-3.0 mL of Resuspension media in the dish, depending on the size of the tumor. It would be advisable to use the smallest amount of media possible (~1.0 mL), which would provide better control during the tissue mincing process.

3. Mince tumor tissue with a sterile surgical scalpel (Size 21) into 0.5-1.0 mm fragments. It would be better to use two scalpel blades and move them in an anti-parallel direction to generate a finely minced tissue preparation. Be careful not to apply too much pressure during this step; if you do, you will end up scraping the plastic from the 35 mm tissue culture plate.

4. Use a P1000 tip to transfer the tissue pulp into a 15.0 mL conical tube. You will not be able to collect all the tissue chunks using a regular P1000 tip, so use a fresh P1000 tip with its end cut to widen the bore. Add 1.0 mL of Resuspension media in a rinsing motion and transfer the digested tissue pulp to the 15.0 mL conical tube. Pellet the tissue suspension at 1500 rpm for 5 minutes at 4℃. Remove the supernatant carefully. Be aware that the tissue pellet will not be firm, so care should be taken not to disturb it. It would be advisable to first remove the majority of the supernatant with a P1000 tip and then use a P200 tip to remove the remaining supernatant.

5. Add 10 mL of ACK lysis buffer to gently resuspend the pellet by inverting the conical tube 5-10 times, incubate at room temperature for 2 minutes to remove red blood cells as head and neck tumor tissues are usually rich with red blood cells. After incubation, centrifuge at 1500 rpm for 5 minutes and remove the supernatant.

6. Repeat step 5 one more time and centrifuge at 1500 rpm for 5 minutes to pellet the tissue.

7. Add 2.0 mL of Digestion media. Using a P1000 tip with a tip cut (for wide bore) gently re-suspend the tissue pellet and transfer the pellet to a fresh 35 mm tissue culture plate. Incubate in 37℃ incubator for no more than 30 minutes. Check every 10 minutes for progress. After about 20

minutes, you should be able to see a distinctive pink/purple hallow around the tissue chunks when observed under a microscope.

8. After 30 minutes, transfer the digested tissue slurry to a 15 mL conical tube. To remove all digested tissues, add an additional 2.0 mL of Resuspension media and transfer to the conical tube using a P1000 tip that has a wide bore, as performed above.

9. Centrifuge at 1500 rpm for 5 minutes at 4℃.

10. Aspirate the supernatant gently using a P1000 tip taking care not to disturb the pellet. Add 2.0 mL of Accutase to the pellet. Mix well using a P1000 tip with a wide bore tip and incubate in a 37℃-water bath or a tissue culture incubator for 30 minutes.

11. During the incubation period, you can prepare a well in a 12-well culture plate for plating the cells. (Coat 1 well of a 12-well plate with 150 µL of Matrigel. To spread it evenly into a single layer, use a P200 pipet tip, and as you release the Matrigel onto the surface of the well, move the tip in zig-zag motion, keeping the space between the lines to a minimum. It is important that you do not eject all the Matrigel during this process, as you will tend to trap air bubbles. As you complete the zig-zag motion from the top to bottom of the well, use the remaining few microliters of Matrigel to coat the circumference of the well with Matrigel. To check for the efficiency of Matrigel coating, you may want to hold the plate towards the light and look for any gaps in the coated surface. Once you are certain that you have evenly coated the Matrigel, move the plates to 37℃ cell culture incubator and leave it there until the cells are ready to be seeded)

12. Remove the cells+Accutase tube from 37℃, add 1.0 mL of Resuspension media. Using a wide bore P1000 tip, gently mix and transfer the tissue slurry to a tissue strainer mounted onto a 50.0 mL conical tube. Move the tip gently over the strainer to allow the digested cells to flow through and tissue debris to remain trapped on top. Add an additional 1.0 mL of Resuspension media to the top of the cell strainer while gently disturbing the debris to release any digested cells that may be trapped.

13. Centrifuge the flow-through at 1500 rpm for 5 minutes at 4℃.

14. Gently resuspend the pellet in 2.0 mL of freshly prepared Culture media. You should not make Culture media with 5.0% Matrigel and let it sit on ice for long periods of time as it will result in sedimentation of the Matrigel. To save time, you may prepare Culture media without Matrigel and keep on ice until ready. Just before you need to resuspend cells, add 5.0% Matrigel and use immediately.

15. Gently, in drops, transfer the 2.0 mL of the resuspended cells to the Matrigel coated well in the 12-well plate. Be careful not to squirt the suspension as you will disturb the underlying Matrigel layer.

16. Monitor growth. You should take representative images every 4 days to document growth. These images can be used to determine the growth rate by morphometric analysis.

17. Changing media. It is important to change media every 3-4 days. As noted earlier, Matrigel containing culture media must be prepared just before addition to the well to avoid sedimentation of the Matrigel. Aspirate the old media gently using a P1000 tip, taking care not to disturb the Matrigel or the organoids. Add Matrigel containing Culture media to the well gently, in drops. Take care not to add it too fast, as it will disturb the organoids and the Matrigel layer.

PROCEDURE FOR MAINTAINING, PASSAGING, AND CRYOPRESERVING ORGANOID CULTURES

- The time to passage the organoids can vary depending on the organoid line. In our experience we have found some PDO lines grow rapidly and are ready for passage by day 12-14, whereas other take much longer. In general, the time to passage is identified by culture that have stopped growing for more than 4 days.

- The protocol below is optimized for one well of an organoid line in a 12-well plate.

1. Aspirate the old media using a pipet tip. Do not use an aspirator. Be careful not to disturb the Matrigel layer or the organoids.

2. Add 0.5 mL/well Digestion media. Add gently in drops, working close to the Matrigel surface to avoid the generation of turbulence by the media being added.

3. Incubate at 37℃ for 1.5 hours. By 1.5 hours, you should be able to observe the Matrigel digested into a pulp/slurry and the organoid dispersed into small clumps or clusters. This would indicate the completion of the digestion. If these changes are not obvious, you could extend the digestion for another 30 minutes.

4. Resuspend the cells by adding 1.0 mL of Resuspension media. The media can be cold, preferably not warmed to 37℃. Add the media in drops. Gently resuspend using a p1000 tip several times until the Matrigel is a smooth suspension and the organoids have dispersed into single cells or smaller clumps. Transfer it to a 15.0 mL conical tube.

5. Centrifuge at 1500 rpm for 5 minutes. The pellet that contains Matrigel and cells will not be prominent and can be only seen as a difference in texture and not necessarily as a difference in color. Remove the supernatant gently using a P100 tip. This is a very critical step as it is very easy to aspirate off the Matrigel/cell pellet. Pay attention to the difference in consistency while removing. To be safe, you may choose to remove the supernatant in two stages: first remove ~1200 µL using P1000 and then remove the rest using a P200 tip. The P200 tip will allow you to readily feel the difference in consistency between the supernatant and the Matrigel/cell pellet.

6. Resuspend the Matrigel pellet by adding 500 µL of Accutase (kept on ice). Accutase comes as 100.00 mL from the vendor. This should be aliquoted into smaller volumes (1.0, 5.0, or 10.0 mL) to avoid repeated freeze-thaw. Pipette up and down until the Matrigel/cell pellet is resuspended into a smooth slurry.

7. Incubate cells+accutase tubes at 37℃ for 20-30 minutes.

8. During the above incubation period, prepare wells in a 12-well plate for re-seeding cells. We typically split the culture at a ratio of 1:2 to 1:4. The choice of split ratio will depend on the growth rate (fast-growing versus slow-growing organoid lines) or other needs for cells (part of the cells to be used for isolating DNA/RNA or for cryopreservation). We have found that some lines grow very well and can be split at a 1:4 ratio, whereas some are slow-growing and require a 1:2 split. It may be wise to perform a 1:2 split during the early passages until you become familiar with the organoid line.

9. Once you determine the split ratio, coat the required number of wells in a 12-well plate with 150 µL of Matrigel. To spread it evenly into a single layer, use a P200 pipet tip, and as you release the Matrigel onto the surface of the well, move the tip in zig-zag motion, keeping the space between the lines to a minimum. It is important that you do not eject all the Matrigel during this process, as you will tend to trap air bubbles. As you complete the zig-zag motion from the top to bottom of the well, use the remaining few microliters of Matrigel to coat the circumference of the well with Matrigel. To check for the efficiency of Matrigel coating, you may want to hold the plate towards the light and look for any gaps in the coated surface. Once you are certain that you have evenly coated the Matrigel, move the plates to 37℃ cell culture incubator and leave it there until the cells are ready to be seeded.

10. Remove the cells+accutase tubes from 37℃ and add 500 µL of Resuspension media. Gently pipette up and down to generate a smooth suspension of cells. Use a small aliquot to determine cell number in a cell counter or hemocytometer. If you need cells for generating a cell pellet (for DNA/RNA isolation), an aliquot of this cell suspension can be used. It is also wise to freeze down (see below for details) as many vials of cells as possible during early passages to have stocks of early passage organoid cultures.

11. Once you have taken all the cells needed for generating a pellet or cryopreservation, pellet the rest of the cells at 1500 rpm for 5.0 minutes at 4℃. Aspirate the media using a P1000 tip and leave a few microliters of media to be safe to not disturb the cell pellet.

For Re-seeding:

12. Prepare Culture media only just before you need and keep it on ice. It would not be advisable to add the 5.0% Matrigel to the media and let it sit for long periods of time as it will result in sedimentation of the Matrigel. You should also bring the Matrigel-coated 12-well plate to the hood, so resuspended cells can be plated immediately.

13. If you are re-seeding a defined number of cells per well, you can re-seed them at a density of 50,000 to 150,000 cells per well in a 12-well plate. The choice of cell number will depend on the organoid line; fast-growing lines can be plated at lower density, whereas slow-growing lines need to be plated at high density. Re-suspend the pellet @ 1.0 mL of Culture media for each well of the 12-well plate. Gently resuspend the cells using a P1000 tip and plate them on the Matrigel-coated well of the 12-well plate immediately.

14. <u>Changing media.</u> It is important to change media every 3-4 days. As noted earlier, Matrigel containing culture media must be prepared just before addition to the well to avoid sedimentation of the Matrigel. You may choose to prepare Culture media without Matrigel but containing ROCK inhibitor and store it on ice until you are ready to add it to the culture dish.

15. Aspirate the old media gently using a P1000 tip, taking care not to disturb the Matrigel or the organoids. Add Matrigel containing Culture media to the well gently, in drops. Take care not to add too fast, as it will disturb the organoids and the Matrigel layer.

16. You should take representative images every four days to document growth. These images can be used to determine the growth rate by morphometric analysis.

For Cryopreservation:

- After removing the supernatant after Accutase digestion, resuspend the pellet with a freezing medium.

- Freeze at 200,000 cells/mL in 500 µL of freezing medium in 1.5 mL Cryovials.

- Store at -80℃ overnight and transfer to a liquid Nitrogen tank the next day.

Supplementary Table S1. Tumor site, stage, adjuvant treatment, and outcomes of patients from whom PDOs were tested for in vitro radiation sensitivity.

Organoid ID	Age (y) / Sex	Location of Specimen	Pathology Diagnosis
**HN-001**	56 / M	Left tonsil tumor<br>Left level 2A lymph node fragment	HPV-mediated squamous cell carcinoma
**HN-002**	58 / F	Right floor of mouth	Squamous cell carcinoma, conventional type, well-differentiated
**HN-003**	83 / M	Left floor of mouth	Squamous cell carcinoma, conventional type, well-differentiated
**HN-004**	52 / F	Left tongue and floor of mouth	Squamous cell carcinoma, conventional type, moderately differentiated
**HN-005**	56 / F	Left tongue base and glossotonsular sulcus	Squamous cell carcinoma, nonkeratinizing (p16-negative)
**HN-007**	66 / M	Level 2A lymph node<br>Oral cavity cancer	Squamous cell carcinoma, conventional type, moderately differentiated
**HN-008**	51 / F	Right neck lymph node mass	Squamous cell carcinoma, p16+ (unknown primary site)
**HN-009**	75 / M	Left ear subcutaneous soft tissue, with extension into left mastoid	Basal cell carcinoma (likely cutaneous origin)
**HN-010**	57 / F	Left tonsillar fossa	HPV-mediated squamous cell carcinoma
**HN-011**	62 / M	Larynx	Squamous cell carcinoma, conventional type, moderately differentiated
**HN-012**	55 / M	Floor of mouth tumor	Squamous cell carcinoma, conventional type, well differentiated
**HN-013**	76 / F	Floor of mouth tumor	Sarcoma (chondrosarcoma)

Organoid ID	Age (y) / Sex	Location of Specimen	Pathology Diagnosis
**HN-014**	54 / M	Larynx	Squamous cell carcinoma, conventional type, moderately differentiated
**HN-015**	52 / M	Larynx	Squamous cell carcinoma, conventional type, moderately differentiated (p16-negative)
**HN-016**	28 / M	Skull base	Olfactory carcinoma
**HN-017**	62 / M	Skull base & maxilla tumor	Sarcoma (undifferentiated pleomorphic sarcoma)
**HN-018**	88 /M	Skin (right cheek)	Cutaneous squamous cell carcinoma
**HN-019**	42 / M	Right oral tongue	Squamous cell carcinoma, conventional type, well differentiated (p16-negative)
**HN-020**	73 / M	Skin (left side of face)	Cutaneous squamous cell carcinoma
**HN-021**	64 /M	Right tonsil	HPV-mediated squamous cell carcinoma
**HN-022**	79 / M	Skin (right side of face below ear)	Cutaneous squamous cell carcinoma
**HN-023**	56 / M	Skin (right side of face)	Cutaneous squamous cell carcinoma
**HN-024**	60 / M	Left tonsil tumor	HPV-mediated squamous cell carcinoma
**HN-025**	51 / M	Soft palate (oral cavity)	Squamous cell carcinoma, conventional type, moderately differentiated
**HN-026**	71 / F	Skull base, ethmoid sinus, maxillary sinus	Sarcoma (biphenotypic sinonasal sarcoma)
**HN-027**	70 / M	Larynx	Squamous cell carcinoma, conventional type, well differentiated

**HN-028**	76 / M	Larynx / pharynx (left piriform sinus)	Squamous cell carcinoma, conventional type, well differentiated
**HN-029**	78 / F	Right buccal (oral cavity)	Squamous cell carcinoma, conventional type, moderately differentiated
**HN-030**	49 / M	Right tonsil	HPV-mediated squamous cell carcinoma
**HN-031**	78 / M	Left parotid and soft tissue of face (recurrence of skin tumor)	Squamous cell carcinoma, conventional type, poorly differentiated
**HN-032**	64 / F	Skin (scalp)	Squamous cell carcinoma, conventional type, moderately to poorly differentiated
**HN-033**	59 / M	Right pharynx	Squamous cell carcinoma, conventional type, moderately differentiated (HPV-negative)
**HN-034**	69 / F	Right buccal (oral cavity)	Squamous cell carcinoma, conventional type, poorly differentiated
**HN-037**	58 / M	Right tonsil	HPV-mediated squamous cell carcinoma

Supplementary Table S2. Patient-derived organoid lines used for experimentation.

Organoid ID	Age (y) / Sex	Location of Specimen	Pathology Diagnosis
**HN-003**	83 / M	Left floor of mouth	Squamous cell carcinoma, conventional type, well-differentiated
**HN-011**	62 / M	Larynx	Squamous cell carcinoma, conventional type, moderately differentiated
**HN-012**	55 / M	Floor of mouth tumor	Squamous cell carcinoma, conventional type, well differentiated
**HN-015**	52 / M	Larynx	Squamous cell carcinoma, conventional type, moderately differentiated (p16-negative)
**HN-018**	88 /M	Skin (right cheek)	Cutaneous squamous cell carcinoma
**HN-021**	64 /M	Right tonsil	HPV-mediated squamous cell carcinoma
**HN-029**	78 / F	Right buccal (oral cavity)	Squamous cell carcinoma, conventional type, moderately differentiated

Abbreviations. M, male; F, female

Supplementary Table S3: Whole exome sequencing variant counts per samples

Sample	Variant Type	Count Level	Counts
HN021 (PDO)	Amplification	Gene	310
HN021 (FFPE)	Amplification	Gene	1334
HN015 (PDO)	Amplification	Gene	12
HN015 (FFPE)	Amplification	Gene	504
HN011 (PDO)	Amplification	Gene	9
HN011 (FFPE)	Amplification	Gene	58
HN003 (PDO)	Amplification	Gene	22
HN003 (FFPE)	Amplification	Gene	132
HN021 (PDO)	Deletion	Gene	13
HN021 (FFPE)	Deletion	Gene	710
HN015 (PDO)	Deletion	Gene	8
HN015 (FFPE)	Deletion	Gene	60
HN011 (PDO)	Deletion	Gene	14
HN011 (FFPE)	Deletion	Gene	25
HN003 (PDO)	Deletion	Gene	11
HN003 (FFPE)	Deletion	Gene	43
HN021 (PDO)	Synonymous	Individual variants	68
HN021 (FFPE)	Synonymous	Individual variants	58
HN015 (PDO)	Synonymous	Individual variants	25
HN015 (FFPE)	Synonymous	Individual variants	30
HN011 (PDO)	Synonymous	Individual variants	39
HN011 (FFPE)	Synonymous	Individual variants	52
HN003 (PDO)	Synonymous	Individual variants	37
HN003 (FFPE)	Synonymous	Individual variants	46
HN021 (PDO)	Missense	Individual variants	167
HN021 (FFPE)	Missense	Individual variants	131
HN015 (PDO)	Missense	Individual variants	65
HN015 (FFPE)	Missense	Individual variants	80
HN011 (PDO)	Missense	Individual variants	74
HN011 (FFPE)	Missense	Individual variants	97
HN003 (PDO)	Missense	Individual variants	80
HN003 (FFPE)	Missense	Individual variants	85
HN021 (PDO)	Stop-gain	Individual variants	9
HN021 (FFPE)	Stop-gain	Individual variants	3
HN015 (PDO)	Stop-gain	Individual variants	4
HN015 (FFPE)	Stop-gain	Individual variants	4
HN011 (PDO)	Stop-gain	Individual variants	1
HN011 (FFPE)	Stop-gain	Individual variants	6
HN003 (PDO)	Stop-gain	Individual variants	2
HN003 (FFPE)	Stop-gain	Individual variants	4

Supplementary Table S4: Whole exome sequencing sample-wise similarity measures

Sample 1	Sample 2	Similarity	Similarity Type	Variant Type
HN021 (FFPE)	HN021 (PDO)	0.636363636	Jaccard	Synonymous
HN003 (FFPE)	HN003 (PDO)	0.62745098	Jaccard	Synonymous
HN011 (FFPE)	HN011 (PDO)	0.568965517	Jaccard	Synonymous
HN015 (FFPE)	HN015 (PDO)	0.447368421	Jaccard	Synonymous
HN003 (FFPE)	HN021 (PDO)	0.085714286	Jaccard	Synonymous
HN011 (PDO)	HN015 (PDO)	0.066666667	Jaccard	Synonymous
HN011 (FFPE)	HN015 (PDO)	0.054794521	Jaccard	Synonymous
HN015 (PDO)	HN021 (FFPE)	0.050632911	Jaccard	Synonymous
HN011 (PDO)	HN015 (FFPE)	0.045454545	Jaccard	Synonymous
HN003 (FFPE)	HN015 (PDO)	0.044117647	Jaccard	Synonymous
HN003 (PDO)	HN021 (FFPE)	0.043956044	Jaccard	Synonymous
HN003 (FFPE)	HN021 (FFPE)	0.04	Jaccard	Synonymous
HN003 (PDO)	HN021 (PDO)	0.03960396	Jaccard	Synonymous
HN011 (FFPE)	HN021 (FFPE)	0.037735849	Jaccard	Synonymous
HN015 (FFPE)	HN021 (FFPE)	0.035294118	Jaccard	Synonymous
HN003 (PDO)	HN011 (FFPE)	0.034883721	Jaccard	Synonymous
HN011 (FFPE)	HN021 (PDO)	0.034482759	Jaccard	Synonymous
HN015 (PDO)	HN021 (PDO)	0.033333333	Jaccard	Synonymous
HN003 (PDO)	HN015 (PDO)	0.033333333	Jaccard	Synonymous
HN011 (PDO)	HN021 (FFPE)	0.031914894	Jaccard	Synonymous
HN003 (FFPE)	HN011 (FFPE)	0.031578947	Jaccard	Synonymous
HN003 (FFPE)	HN015 (FFPE)	0.027027027	Jaccard	Synonymous
HN011 (FFPE)	HN015 (FFPE)	0.025	Jaccard	Synonymous
HN003 (FFPE)	HN011 (PDO)	0.024096386	Jaccard	Synonymous
HN015 (FFPE)	HN021 (PDO)	0.020833333	Jaccard	Synonymous
HN011 (PDO)	HN021 (PDO)	0.019047619	Jaccard	Synonymous
HN003 (PDO)	HN015 (FFPE)	0.015151515	Jaccard	Synonymous
HN003 (PDO)	HN011 (PDO)	0.013333333	Jaccard	Synonymous
HN021 (FFPE)	HN021 (PDO)	0.712643678	Jaccard	Missense
HN003 (FFPE)	HN003 (PDO)	0.633663366	Jaccard	Missense
HN015 (FFPE)	HN015 (PDO)	0.510416667	Jaccard	Missense
HN011 (FFPE)	HN011 (PDO)	0.5	Jaccard	Missense
HN003 (PDO)	HN015 (PDO)	0.074074074	Jaccard	Missense
HN003 (FFPE)	HN021 (PDO)	0.058823529	Jaccard	Missense
HN003 (FFPE)	HN015 (PDO)	0.056338028	Jaccard	Missense
HN015 (PDO)	HN021 (PDO)	0.049773756	Jaccard	Missense
HN003 (PDO)	HN021 (PDO)	0.046610169	Jaccard	Missense
HN011 (FFPE)	HN015 (PDO)	0.04516129	Jaccard	Missense
HN003 (PDO)	HN021 (FFPE)	0.044554455	Jaccard	Missense
HN015 (PDO)	HN021 (FFPE)	0.042553191	Jaccard	Missense
HN003 (PDO)	HN011 (FFPE)	0.041176471	Jaccard	Missense
HN003 (FFPE)	HN011 (FFPE)	0.04	Jaccard	Missense
HN003 (PDO)	HN015 (FFPE)	0.038961039	Jaccard	Missense
HN003 (FFPE)	HN021 (FFPE)	0.038461538	Jaccard	Missense
HN011 (PDO)	HN015 (PDO)	0.037313433	Jaccard	Missense
HN003 (PDO)	HN011 (PDO)	0.033557047	Jaccard	Missense
HN003 (FFPE)	HN015 (FFPE)	0.03125	Jaccard	Missense

HN011 (FFPE)	HN021 (PDO)	0.027237354	Jaccard	Missense
HN011 (FFPE)	HN021 (FFPE)	0.027027027	Jaccard	Missense
HN011 (PDO)	HN015 (FFPE)	0.026666667	Jaccard	Missense
HN003 (FFPE)	HN011 (PDO)	0.025806452	Jaccard	Missense
HN015 (FFPE)	HN021 (PDO)	0.020661157	Jaccard	Missense
HN015 (FFPE)	HN021 (FFPE)	0.019323671	Jaccard	Missense
HN011 (FFPE)	HN015 (FFPE)	0.017241379	Jaccard	Missense
HN011 (PDO)	HN021 (PDO)	0.016877637	Jaccard	Missense
HN011 (PDO)	HN021 (FFPE)	0.009852217	Jaccard	Missense
HN015 (FFPE)	HN015 (PDO)	1	Jaccard	Stop-gain
HN021 (FFPE)	HN021 (PDO)	0.333333333	Jaccard	Stop-gain
HN003 (FFPE)	HN003 (PDO)	0.2	Jaccard	Stop-gain
HN011 (FFPE)	HN011 (PDO)	0.166666667	Jaccard	Stop-gain
HN003 (FFPE)	HN021 (PDO)	0.083333333	Jaccard	Stop-gain
HN003 (PDO)	HN021 (PDO)	0	Jaccard	Stop-gain
HN011 (FFPE)	HN021 (PDO)	0	Jaccard	Stop-gain
HN011 (PDO)	HN021 (PDO)	0	Jaccard	Stop-gain
HN015 (FFPE)	HN021 (PDO)	0	Jaccard	Stop-gain
HN015 (PDO)	HN021 (PDO)	0	Jaccard	Stop-gain
HN003 (FFPE)	HN021 (FFPE)	0	Jaccard	Stop-gain
HN003 (PDO)	HN021 (FFPE)	0	Jaccard	Stop-gain
HN011 (FFPE)	HN021 (FFPE)	0	Jaccard	Stop-gain
HN011 (PDO)	HN021 (FFPE)	0	Jaccard	Stop-gain
HN015 (FFPE)	HN021 (FFPE)	0	Jaccard	Stop-gain
HN015 (PDO)	HN021 (FFPE)	0	Jaccard	Stop-gain
HN003 (FFPE)	HN015 (PDO)	0	Jaccard	Stop-gain
HN003 (PDO)	HN015 (PDO)	0	Jaccard	Stop-gain
HN011 (FFPE)	HN015 (PDO)	0	Jaccard	Stop-gain
HN011 (PDO)	HN015 (PDO)	0	Jaccard	Stop-gain
HN003 (FFPE)	HN015 (FFPE)	0	Jaccard	Stop-gain
HN003 (PDO)	HN015 (FFPE)	0	Jaccard	Stop-gain
HN011 (FFPE)	HN015 (FFPE)	0	Jaccard	Stop-gain
HN011 (PDO)	HN015 (FFPE)	0	Jaccard	Stop-gain
HN003 (FFPE)	HN011 (PDO)	0	Jaccard	Stop-gain
HN003 (PDO)	HN011 (PDO)	0	Jaccard	Stop-gain
HN003 (FFPE)	HN011 (FFPE)	0	Jaccard	Stop-gain
HN003 (PDO)	HN011 (FFPE)	0	Jaccard	Stop-gain
HN015 (FFPE)	HN015 (PDO)	1	Szymkiewicz–Simpson	Amplification
HN021 (FFPE)	HN021 (PDO)	0.985266086	Szymkiewicz–Simpson	Amplification
HN011 (FFPE)	HN011 (PDO)	0.768303061	Szymkiewicz–Simpson	Amplification
HN003 (FFPE)	HN003 (PDO)	0.577384314	Szymkiewicz–Simpson	Amplification
HN003 (FFPE)	HN021 (PDO)	0.301908414	Szymkiewicz–Simpson	Amplification
HN003 (FFPE)	HN021 (FFPE)	0.301908414	Szymkiewicz–Simpson	Amplification
HN003 (FFPE)	HN015 (FFPE)	0.08497471	Szymkiewicz–Simpson	Amplification
HN003 (PDO)	HN021 (PDO)	0	Szymkiewicz–Simpson	Amplification
HN011 (FFPE)	HN021 (PDO)	0	Szymkiewicz–Simpson	Amplification
HN011 (PDO)	HN021 (PDO)	0	Szymkiewicz–Simpson	Amplification
HN015 (FFPE)	HN021 (PDO)	0	Szymkiewicz–Simpson	Amplification
HN015 (PDO)	HN021 (PDO)	0	Szymkiewicz–Simpson	Amplification
HN003 (PDO)	HN021 (FFPE)	0	Szymkiewicz–Simpson	Amplification

HN011 (FFPE)	HN021 (FFPE)	0	Szymkiewicz–Simpson	Amplification
HN011 (PDO)	HN021 (FFPE)	0	Szymkiewicz–Simpson	Amplification
HN015 (FFPE)	HN021 (FFPE)	0	Szymkiewicz–Simpson	Amplification
HN015 (PDO)	HN021 (FFPE)	0	Szymkiewicz–Simpson	Amplification
HN003 (FFPE)	HN015 (PDO)	0	Szymkiewicz–Simpson	Amplification
HN003 (PDO)	HN015 (PDO)	0	Szymkiewicz–Simpson	Amplification
HN011 (FFPE)	HN015 (PDO)	0	Szymkiewicz–Simpson	Amplification
HN011 (PDO)	HN015 (PDO)	0	Szymkiewicz–Simpson	Amplification
HN003 (PDO)	HN015 (FFPE)	0	Szymkiewicz–Simpson	Amplification
HN011 (FFPE)	HN015 (FFPE)	0	Szymkiewicz–Simpson	Amplification
HN011 (PDO)	HN015 (FFPE)	0	Szymkiewicz–Simpson	Amplification
HN003 (FFPE)	HN011 (PDO)	0	Szymkiewicz–Simpson	Amplification
HN003 (PDO)	HN011 (PDO)	0	Szymkiewicz–Simpson	Amplification
HN003 (FFPE)	HN011 (FFPE)	0	Szymkiewicz–Simpson	Amplification
HN003 (PDO)	HN011 (FFPE)	0	Szymkiewicz–Simpson	Amplification
HN003 (FFPE)	HN003 (PDO)	0.875476624	Szymkiewicz–Simpson	Deletion
HN015 (FFPE)	HN015 (PDO)	0.071695854	Szymkiewicz–Simpson	Deletion
HN015 (PDO)	HN021 (FFPE)	0.005002296	Szymkiewicz–Simpson	Deletion
HN003 (FFPE)	HN021 (PDO)	0	Szymkiewicz–Simpson	Deletion
HN003 (PDO)	HN021 (PDO)	0	Szymkiewicz–Simpson	Deletion
HN011 (FFPE)	HN021 (PDO)	0	Szymkiewicz–Simpson	Deletion
HN011 (PDO)	HN021 (PDO)	0	Szymkiewicz–Simpson	Deletion
HN015 (FFPE)	HN021 (PDO)	0	Szymkiewicz–Simpson	Deletion
HN015 (PDO)	HN021 (PDO)	0	Szymkiewicz–Simpson	Deletion
HN021 (FFPE)	HN021 (PDO)	0	Szymkiewicz–Simpson	Deletion
HN003 (FFPE)	HN021 (FFPE)	0	Szymkiewicz–Simpson	Deletion
HN003 (PDO)	HN021 (FFPE)	0	Szymkiewicz–Simpson	Deletion
HN011 (FFPE)	HN021 (FFPE)	0	Szymkiewicz–Simpson	Deletion
HN011 (PDO)	HN021 (FFPE)	0	Szymkiewicz–Simpson	Deletion
HN015 (FFPE)	HN021 (FFPE)	0	Szymkiewicz–Simpson	Deletion
HN003 (FFPE)	HN015 (PDO)	0	Szymkiewicz–Simpson	Deletion
HN003 (PDO)	HN015 (PDO)	0	Szymkiewicz–Simpson	Deletion
HN011 (FFPE)	HN015 (PDO)	0	Szymkiewicz–Simpson	Deletion
HN011 (PDO)	HN015 (PDO)	0	Szymkiewicz–Simpson	Deletion
HN003 (FFPE)	HN015 (FFPE)	0	Szymkiewicz–Simpson	Deletion
HN003 (PDO)	HN015 (FFPE)	0	Szymkiewicz–Simpson	Deletion
HN011 (FFPE)	HN015 (FFPE)	0	Szymkiewicz–Simpson	Deletion
HN011 (PDO)	HN015 (FFPE)	0	Szymkiewicz–Simpson	Deletion
HN003 (FFPE)	HN011 (PDO)	0	Szymkiewicz–Simpson	Deletion
HN003 (PDO)	HN011 (PDO)	0	Szymkiewicz–Simpson	Deletion
HN011 (FFPE)	HN011 (PDO)	0	Szymkiewicz–Simpson	Deletion
HN003 (FFPE)	HN011 (FFPE)	0	Szymkiewicz–Simpson	Deletion
HN003 (PDO)	HN011 (FFPE)	0	Szymkiewicz–Simpson	Deletion
