# Supplementary material for: Head and neck tumor organoid grown under simplified media conditions model tumor biology and chemoradiation responses
Source: Sci Rep. 2025 Jul 7;15:24221. doi: 10.1038/s41598-025-88082-5 (PMC12234821; doi:10.1038/s41598-025-88082-5)
Supplement: Supplementary file 2 — Supplementary Material 2 [file 41598_2025_88082_MOESM2_ESM.pdf]

Supplementary Table S5

**Recipe**	Our Manuscript	Recipe 2 [1,2]	Recipe 3 [3]	Recipe 4 [4]	Recipe 5 [5]
Advanced DMEM/F12	✓	✓	✓	✓	✓
GlutaMAX		1✕	1✕		1%
Penicillin-streptomycin	1%	1%	1%		1%
HEPES		10 mM	1✕		
B27	1.0 mL for 100 mL	1✕	1✕	1✕	1✕
N-acetyl-L-cysteine		1.25 mM	0.1 mM	1 mM (only used during processing)	1.25 mM
Nicotinamide		10 mM	10 nM		10 mM
human EGF	10 ng/mL	50 ng/mL	50 ng/mL	50 ng/mL	50 ng/mL
A83-01		500 nM	500 nM		500 nM
human FGF10	10 ng/mL	10 ng/mL			10 ng/mL
human FGF2	10 ng/mL	5 ng/mL		20 ng/ mL	5 ng/mL
Prostaglandin E2		1 µM			1 µM
CHIR 99021		0.3 µM			0.3 µM
Forskolin		1 µM			1 µM
R-spondin		4%	✓	✓	
Noggin		4%	✓	✓	✓
Y-27632	10 µM	10 µM	10 µM	10 µM	10 µM
Gastrin			10 nM	10nM (only used during processing)	
SB202190			10 nM		
Recombinant human Wnt3A			100 ng/mL	✓	✓
N2			1✕	1✕	
Hydrocortisone	0.5 µg/mL				
Cholerae Toxin	200 ng/mL				
Human Insulin	10 µg/mL				
Recombinant Human Amphiregulin	50 ng/mL			250 µg/mL (only first day of culture)	
Recombinant Human Prolactin	10 ng/mL				
Recombinant Human IL6	100 ng/mL				
Bovine Pituitary Extract (BPE)	0.8 mL for 100 mL				
TGF-ß RI kinase-inhibitor VI, SB431542				0.5 µM	
Primocin	100 µg/mL				100 µg/mL
Conditioned media			2% Noggin/R-Spondin-conditioned media	1% Wnt-3A, R-spondin and Noggin-conditioned medium from cell line CRL-2376™	50% Wnt3a, RSPO3, Noggin-conditioned media<br>10% RSPO1-conditioned media

References:

1 Haughton, P. D. *et al*. Differential transcriptional invasion signatures from patient derived organoid models define a functional prognostic tool for head and neck cancer. *Oncogene* **43**, 2463-2474, doi:10.1038/s41388-024-03091-4 (2024).

2 de Kort, W. W. B. *et al*. Clinicopathological Factors as Predictors for Establishment of Patient Derived Head and Neck Squamous Cell Carcinoma Organoids. *Head Neck Pathol* **18**, 59, doi:10.1007/s12105-024-01658-x (2024).

3 Kijima, T. *et al*. Three-Dimensional Organoids Reveal Therapy Resistance of Esophageal and Oropharyngeal Squamous Cell Carcinoma Cells. *Cell Mol Gastroenterol Hepatol* **7**, 73-91, doi:10.1016/j.jcmgh.2018.09.003 (2019).

4 Fisch, A. S. *et al*. Feasibility analysis of using patient-derived tumour organoids for treatment decision guidance in locally advanced head and neck squamous cell carcinoma. *Eur J Cancer* **213**, 115100, doi:10.1016/j.ejca.2024.115100 (2024).

5 Perreard, M. *et al*. ORGAVADS: establishment of tumor organoids from head and neck squamous cell carcinoma to assess their response to innovative therapies. *BMC Cancer* **23**, 223, doi:10.1186/s12885-023-10692-x (2023).
